# A Crucial Role for the Protein Quality Control System in Motor Neuron Diseases

**DOI:** 10.3389/fnagi.2020.00191

**Published:** 2020-07-21

**Authors:** Riccardo Cristofani, Valeria Crippa, Maria Elena Cicardi, Barbara Tedesco, Veronica Ferrari, Marta Chierichetti, Elena Casarotto, Margherita Piccolella, Elio Messi, Mariarita Galbiati, Paola Rusmini, Angelo Poletti

**Affiliations:** ^1^Laboratorio di Biologia Applicata, Dipartimento di Scienze Farmacologiche e Biomolecolari, Dipartimento di Eccellenza 2018-2022, Università degli Studi di Milano, Milan, Italy; ^2^Department of Neuroscience, Jefferson Weinberg ALS Center, Vickie and Jack Farber Institute for Neuroscience, Sidney Kimmel Medical College, Thomas Jefferson University, Philadelphia, PA, United States; ^3^Center of Excellence on Neurodegenerative Diseases (CEND), Università degli Studi di Milano, Milan, Italy

**Keywords:** motor neuron, protein quality control, CASA complex, HSPB8, BAG3, BAG1

## Abstract

Motor neuron diseases (MNDs) are fatal diseases characterized by loss of motor neurons in the brain cortex, in the bulbar region, and/or in the anterior horns of the spinal cord. While generally sporadic, inherited forms linked to mutant genes encoding altered RNA/protein products have also been described. Several different mechanisms have been found altered or dysfunctional in MNDs, like the protein quality control (PQC) system. In this review, we will discuss how the PQC system is affected in two MNDs—spinal and bulbar muscular atrophy (SBMA) and amyotrophic lateral sclerosis (ALS)—and how this affects the clearance of aberrantly folded proteins, which accumulate in motor neurons, inducing dysfunctions and their death. In addition, we will discuss how the PQC system can be targeted to restore proper cell function, enhancing the survival of affected cells in MNDs.

## Introduction

Motor neuron diseases (MNDs) are neurodegenerative diseases (NDs) characterized by the loss of motor neurons in the brain cortex, in the bulbar region, and/or in the anterior horns of the spinal cord; the consequence of motor neuron death is the lack of control on the skeletal muscle fibers. While motor neurons are considered the primary target in MNDs, muscle and glial cells may also be directly involved, and this affects motor neuron survival. MNDs are generally fatal diseases, clinically characterized by severe loss of voluntary movements, muscle weakness, spasticity, and atrophy. MNDs appear as sporadic or inherited forms, which have been extensively studied in the last 30 years. The inherited forms are associated with gene mutations that result in the production of altered RNA or proteins with reduced [loss-of-function (LOF)] or aberrant neurotoxic [gain-of-function (GOF)] functions. Mixed LOF and GOF are also possible. In LOF, the RNA or the protein affected are generally essential for motor neuron viability; thus, their reduced activity often causes motor neuron death [e.g., in spinal muscular atrophy (SMA); Lefebvre et al., 1995]. In these cases, the therapeutic intervention is aimed to restore the proper activity of the missed/altered RNA or protein (Poletti and Fischbeck, 2020), and successful therapies have been recently approved worldwide from regulatory agencies (Finkel et al., 2017; Mendell et al., 2017; Mercuri et al., 2018). In GOF, different neurotoxic mechanisms have been reported to take place in a given mutant RNA or protein. Unfortunately, this makes difficult to identify a common therapeutic target for MNDs. Therefore, these approaches must be specifically designed for each MND’s form. However, it is now clear that many familial MND forms are characterized by alterations of common intracellular pathways, which are often also altered in sporadic MNDs. Thus, these pathways might serve as potential therapeutic targets to reduce motor neuron death. In this review, we will focus on one of the most common pathways affected in MNDs, the protein quality control (PQC) system. In fact, in several MNDs, which include spinal and bulbar muscular atrophy (SBMA) and amyotrophic lateral sclerosis (ALS), the PQC system becomes unable to correctly handle misfolded proteins (mainly produced by the mutant gene), letting them become harmful to motor neurons and/or to glial and skeletal muscle cells.

## Misfolded Proteins Associated With Motor Neuron Diseases

### Spinal and Bulbar Muscular Atrophy

SBMA is the first MND for which a specific gene mutation has been linked to the disease as the cause of neuronal cell death (La Spada et al., 1991). SBMA, initially defined as a pure MND, is presently also classified as a neuromuscular disease. In fact, in SBMA, the primarily affected cell populations are lower motor neurons localized in the bulbar region of the brain (brain stem containing motor neurons of the lower cranial nerves) or in the anterior horn of the spinal cord (Sobue et al., 1989; La Spada et al., 1991; Brooks and Fischbeck, 1995; Li et al., 1995; Brooks et al., 1997). Dorsal root ganglia (DRG) neurons may also be affected in SBMA (Chua and Lieberman, 2013) and the combination of motor and DRG neurons loss is responsible for the clinical signs which include muscle fasciculations, weakness, and subsequent atrophy, including dysphagia and dysarthria with atrophy of the bulbar, facial, and limb muscles, as well as sensory disturbances at distal extremities (Sobue et al., 1989). So far, there is no evidence for the involvement of other brain cell types (e.g., glial cells or microglia). In addition to neuronal cells, skeletal muscle cells are also directly affected in SBMA (Chua and Lieberman, 2013; Cortes et al., 2014a; Lieberman et al., 2014; Rinaldi et al., 2014; Rusmini et al., 2015; Cicardi et al., 2019). This specific cell susceptibility is because the gene responsible for SBMA encodes for the androgen receptor (AR), and this gene is highly expressed in all the cell types described above (Poletti, 2004; Marron et al., 2005). The same cells express high levels of androgen-activating enzymes (Poletti et al., 1994, 1997, 2001; Pozzi et al., 2003). SBMA patients show mild endocrine alterations, like hypogonadism, possibly due to modification of the gonadal-hypothalamic axis or gynecomastia (Sobue et al., 1989; Kazemi-Esfarjani et al., 1995; Polo et al., 1996; Belsham et al., 1998; Piccioni et al., 2001). These alterations are often associated with reduced AR function.

Since the AR gene locus is on the X-chromosome, SBMA exists only as X-linked inherited form, but only males are affected (La Spada et al., 1991). Notably, the mutated AR protein is inactive in the absence of androgens [testosterone or its derivative 5α-dihydrotestosterone (DHT)], while it acquires toxic properties upon agonist binding (Katsuno et al., 2002, 2003), and the presence of androgens is thus mandatory for symptoms appearance and disease manifestation. This is possible since the AR mutation found in SBMA is radically different from those responsible for partial or complete androgen insensitivity syndrome (PAIS or CAIS) or tumors like prostate cancer (Brinkmann, 2001). In SBMA, the mutant AR gene is characterized by an expansion of a CAG (cytosine, adenine, guanine) tandem repeat (La Spada et al., 1991). The CAG sequence is expressed in exon 1 of the mRNA and then translated into a polyglutamine tract in the AR N-terminus (ARpolyQ). In normal individuals, the polyQ length of AR is highly polymorphic, ranging from 15 to 35 Qs (Edwards et al., 1992; Kuhlenbäumer et al., 2001); in SBMA patients the polyQ size becomes longer than 37 Qs (to a maximum of 72; Fischbeck, 1997; Kuhlenbäumer et al., 2001; Grunseich et al., 2014; Madeira et al., 2018). CAG repeat expansions coding for elongated polyQ tracts have been found in other eight genes, which are unrelated to AR; the mutant protein products of these genes cause other similar NDs (Ross, 2002). The ARpolyQ retains approximately 30% of its transcriptional functions, which explains the endocrine signs present in SBMA, but acquires a novel toxic function that impacts neuronal and muscle cell viability. As mentioned above, this toxic function of ARpolyQ appears after its activation by androgens. These AR ligands (testosterone or DHT) may induce aberrant protein conformations to ARpolyQ (protein misfolding), which becomes highly prone to aggregation (Stenoien et al., 1999; Simeoni et al., 2000; Piccioni et al., 2002). Details of this pathological mechanism are provided below.

### Amyotrophic Lateral Sclerosis

ALS is a typical MND characterized by the loss of both the cerebral motor cortex or brainstem (upper) motor neurons and the cranial nerves and ventral horns of the spinal cord (lower) motor neurons. Neurons located in the frontotemporal cortex may be involved in some specific forms of ALS (Robberecht and Philips, 2013), which may clinically manifest in a pure MND form or be associated with a different extension to frontotemporal dementia (ALS-FTD). Differently from SBMA, the surrounding non-neuronal glial cells [astrocytes (Trotti et al., 1999; Boillee et al., 2006; Nagai et al., 2007), oligodendrocytes (Philips et al., 2013), and Schwann cells (Lobsiger et al., 2009; Manjaly et al., 2010)] are indirectly or directly affected in ALS. Reactive microglia are also present in ALS-affected tissues, but not in SBMA (Philips and Robberecht, 2011), proving that neuroinflammation and oxidative stress may play a significant role in ALS (Ferraiuolo et al., 2011). As in SBMA, the striatal skeletal muscle target cells can also be directly affected in ALS (Dobrowolny et al., 2008; Onesto et al., 2011; Cicardi et al., 2018; Meroni et al., 2019). Ninety percent of ALS cases appear as sporadic (sALS) forms, and only 10% of cases are caused by inherited mutations linked to familial (fALS) forms. The two types of ALS are clinically indistinguishable. Up to now, more than 30 genes have been found altered in fALS (Robberecht and Philips, 2013; Cook and Petrucelli, 2019; Mathis et al., 2019; Mejzini et al., 2019), and each of these accounts for disease, which mainly occurs as monogenic disease, even if disease modifier genes might exist. It is noteworthy that several of the gene products that cause a specific fALS have been reported to acquire an aberrant behavior of their wild-type (wt) forms in sALS. This suggests the existence of common pathways that lead to motor neuronal death in both fALS and in sALS (Neumann et al., 2006; Daoud et al., 2009; Bosco and Landers, 2010).

ALS has a very high variability in terms of both age of onset and disease progression, and it seems to occur earlier in males compared to females (Vegeto et al., 2020), with a male/female ratio of 1–3 in the geographic region and population evaluated in the study (Kurtzke, 1982; Haverkamp et al., 1995; Manjaly et al., 2010). The two sexes also show different symptomatology, since in males the disease predominantly begins in the lumbar tract of the spinal cord, while in females ALS mainly begins in the bulbar region (see Blasco et al., 2012 for an extensive review). It is likely that hormonal sex steroids may influence the neurotoxicity of factors involved in the pathogenesis of ALS (see Vegeto et al., 2020 for an extensive review).

Historically, the *superoxide dismutase 1 (SOD1)* gene is the first gene associated with fALS. However, this mutation only accounts for 15% of all fALS cases. *SOD1* encodes a ubiquitously-expressed antioxidant enzyme that acts as a free radical scavenger enzyme (Bendotti et al., 2012). The most frequent fALS form (almost 50% of all fALS) is due to a mutation in the *C9orf72* (chromosome 9 open reading frame 72) gene; in particular, the mutation consists of an expansion of a hexanucleotide (G_4_C_2_) repeat located in the 5′-untranslated region of the *C9orf72* gene. Surprisingly, despite its location in an intronic sequence, the G_4_C_2_ expansion (which is transcribed in both directions) is utilized by ribosomes as a starting point for translation; this results in the production of five different dipeptides (DPRs; Ash et al., 2013; Gendron et al., 2013; Lashley et al., 2013; Mori et al., 2013). The process has been identified as an unconventional translation and named “repeat-associated non-ATG (RAN) translation” (Zu et al., 2011). The five DPRs do not have a physiological role, but they only exert toxicity in the expressing cells of affected individuals. Other mutant genes are less frequently represented in fALS: examples are the genes encoding TAR DNA-binding protein 43 (TDP-43), the ALS-linked fused in sarcoma/translocated in liposarcoma (FUS/TLS), the ubiquilin-2, the optineurin, the valosin-containing protein/p97 (VCP/p97), and others. These alterations occur in a few fALS families, but the same proteins (even if in the wt form) can be dysregulated in sALS, suggesting that their functions are crucial to maintain neuronal homeostasis (a list of the most common gene mutations identified so far in fALS is reported in Table 1). In particular, TDP-43 is considered a hallmark for sALS since it mislocalizes from nucleus to cytoplasm, where it aggregates in inclusions. These inclusions are enriched by TDP-43 caspase-3-cleaved fragments containing the C-terminal unstructured domain (Ratti and Buratti, 2016).

**Table 1:** Gene mutations reported in familial amyotrophic lateral sclerosis (fALS).

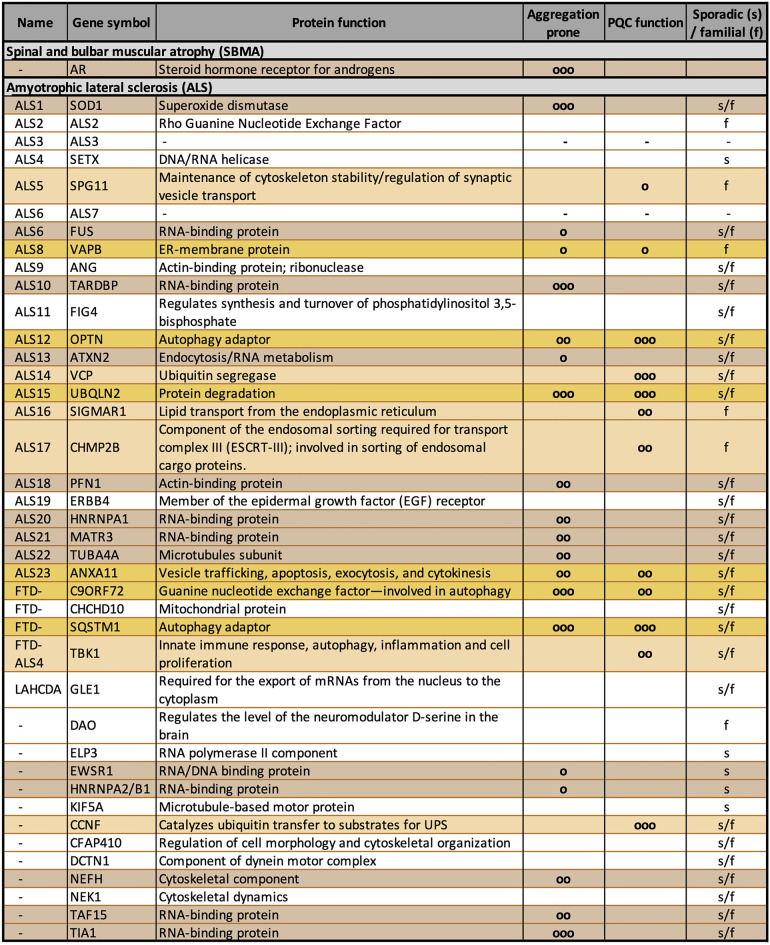

*The table shows the list of the most common mutated genes identified in fALS. The columns describe ALS name, gene symbol, protein function, aggregation propensity, involvement in PQC and sporadic vs. familial form (**o** = low; **oo** = mid; **ooo** = high). Aggregation prone proteins are highlighted in brown, proteins involved in PQC system in orange, and those that show both conditions in yellow*.

A careful analysis of the gene products identified so far suggests that several of their coded proteins have functions that cluster in specific intracellular processes. One of the most represented pathways is the PQC system (Table 1). In fact, different ALS-associated proteins are directly involved in the PQC system and others indirectly affect the PQC system due to their mutation. Indeed, when mutated, they become unable to properly reach the folded conformation and misfold. Misfolded proteins must be cleared from cells, and with this mechanism they may overwhelm the PQC system capability to handle proteotoxic stresses. As in the case of ARpolyQ and in all other elongated polyQ-containing proteins, which cause adult-onset MNDs, the misfolded ALS proteins tend to segregate from the nuclear or cytoplasmic compartments *via* a liquid-liquid phase partitioning (Molliex et al., 2015; Patel et al., 2015; Ganassi et al., 2016; Lee et al., 2016; Alberti et al., 2017; Boeynaems et al., 2017; Freibaum and Taylor, 2017; Mackenzie et al., 2017). This leads to an initial seed of aggregates with well-defined physical-chemical properties, which then mature into aggresomes and insoluble inclusions (Davies et al., 1997; DiFiglia et al., 1997; Li et al., 1998; Lieberman et al., 1998; Kopito, 2000; Mediani et al., 2019). The accumulating proteins may thus damage the PQC system by saturating its functional capabilities or by clogging the pathways devoted to protein clearance. For these reasons, by forming aggregates, misfolded ARpolyQ or ALS-associated proteins may perturb not only the PQC system, but also a series of pathways that depend on the proper functioning of the PQC system to maintain the correct cellular homeostasis.

## The Protein Quality Control System

Most cell types affected in MNDs are post-mitotic or generally characterized by a poor mitotic index. This means that these cells might accumulate aberrant proteins that cannot be diluted by cell self-renewal or by simple partitioning into duplicated intracellular compartments generated as a result of cell division. Thus, these cells must develop a very sophisticated system to maintain their proper protein homeostasis. Therefore, post-mitotic non-dividing cells like neurons, motor neurons, or skeletal muscle cells, as well as poorly replicating cells, like glial cells, are highly prone to respond to misfolded protein species. Misfolded species may be produced in response to different cell stresses or as a consequence of gene mutations. These cells are able to respond to these stresses in a very powerful way: the overexpression of specific chaperones and co-chaperones, paralleled by the potentiation of the degradative pathways. All these factors are extremely well-coordinated to protect against proteotoxicity, and their synergic activities constitute the PQC system mentioned above. The PQC system thus acts as the first line of defense and because of its protective action, its selective modulation represents a valuable target for therapeutic intervention in all protein misfolding diseases, including MNDs like SBMA and ALS.

The PQC system is composed of a very large number of factors clustered in specific families of proteins that work together to define the fate of every single protein starting from its proper folding after synthesis or denaturation, and it routes proteins to degradation in case the folding fails.

### The Chaperones

**Figure 1:**
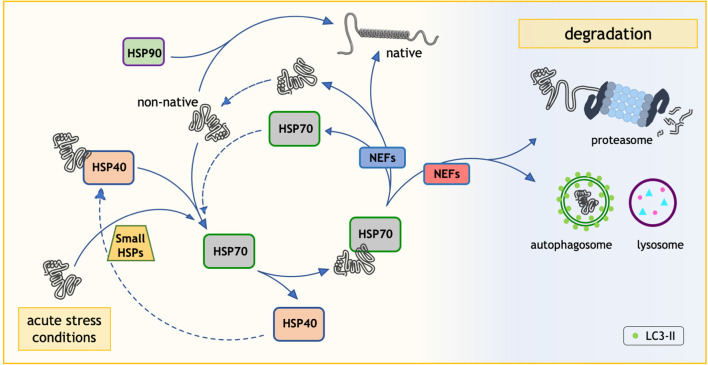
The role of heat shock proteins (HSP70) in the protein quality control (PQC) system. HSP70 plays an essential role in the protein folding process. Through its interaction with HSP40, HSP70 is able to fold the proteins in non-native conformations. HSP70 and HSP40 are not the only HSPs involved. In fact, the HSP90 system can assist protein folding in an independent way and the small HSPs respond to acute stress conditions. Notably, when necessary nucleotide exchange factors (NEFs/BAGs) route HSP70 client proteins to degradative pathways (Ubiquitin-proteasome system and/or autophagy).

The family of intracellular chaperones and their co-chaperones is composed of more than 180 different proteins, some of which share a high degree of homology. These chaperones generally act in a specific subcellular compartment: for example, some chaperones localize exclusively in the endoplasmic reticulum, in the mitochondria, in the lysosomes, and/or in the cytoplasm, where they mainly exert their protective activities. Most chaperones are also expressed in a cell- and tissue-specific manner, with some chaperones localized exclusively in one tissue (e.g., in the testis), while others are ubiquitously expressed. In addition, chaperones may be regulated in response to cell stresses. Indeed, chaperones have been discovered as proteins induced by heat shock, and found to protect cells against thermal damages. Because of this, they have been named “heat shock proteins” or HSPs (DiDomenico et al., 1982). This name still stands for many chaperones, even if they have been demonstrated to possess much wider activities against a spectrum of variables capable of damaging intracellular proteins (e.g., oxidative stress, hypoxia, DNA damage, aberrant translation, etc.). Based on their structure and functions, these factors have been classified in subfamilies of chaperones. Originally, chaperones were grouped based on their apparent molecular weight after their biochemical identification in SDS-PAGE (small HSPs, HSP40s, HSP60s, HSP70s, HSP90s, and HSP100), but this classification now reflects their functions in the folding processes. Based on HUGO Gene Nomenclature Committee, a new nomenclature has been adopted for the human HSP families: HSPB (small HSP), DNAJ (HSP40), HSPD (HSP60), HSPA (HSP70), HSPC (HSP90), and HSPH (HSP110; Kampinga et al., 2009; see also Kampinga and Craig, 2010) for an extensive review). Chaperones often require the assistance of co-chaperones, which serve as nucleotide exchange factors (NEFs), like the members of the BCL2-associated athanogene (BAG) protein family (Takayama and Reed, 2001; Figure 1).

### The Degradative Systems

Cells, including post-mitotic cells like neurons and skeletal muscle cells, utilize two major degradative systems to enzymatically destroy aberrant proteinaceous materials and recycle their components for other proteins production. This process is assisted by chaperones (and their co-chaperones), which route aberrant proteins to degradative systems.

Proteins undergoing this degradation are damaged proteins or regulatory proteins that ended their functions. The two degradative systems are: (a) the ubiquitin proteasome system (UPS); and (b) the autophago-lysosomal pathway (ALP). Of note, UPS acts both in the cytosolic and nuclear compartment, while ALP acts only in the cell cytoplasm.

(a) The UPS is a highly specific and very selective proteolytic system mainly devoted to the clearance of short-lived proteins. The UPS inactivates proteins controlling cell cycle progression, apoptosis, transcription, and cell differentiation. Moreover, the UPS mediates the immune response and it is responsible for the clearance of damaged monomeric proteins. UPS is based on two subsequent steps: the protein is labeled by a covalent binding to ubiquitin (a small protein of 76 amino acids), which is itself ubiquitinated forming a poly-ubiquitin chain of several molecules of ubiquitin (Pickart, 2001a,b); and this poly-ubiquitinated protein is degraded by the 26S proteasome. The recognition of the protein to be degraded is mediated by different chaperones of the HSP70/HSP40 families in a complex (Figure 1). HSP70 has the ability to interact with specific E3-ubiquitin ligases (such as the C-terminus HSP70 interacting protein, CHIP), which selectively ubiquitinate misfolded proteins (Ciechanover, 1994; Ciechanover and Brundin, 2003; Ciechanover and Kwon, 2015). The ubiquitination cascade is rather complex. Ubiquitination initially requires the activation of E1 enzymes that activate ubiquitin; next, the activated ubiquitin is transferred to E2 enzymes, which in concert with the E3-ubiquitin ligases bind ubiquitin to a lysine residue of the substrate protein. E3-ubiquitin ligases have slightly different functions (Jackson et al., 2000; Joazeiro and Weissman, 2000). In addition, deubiquitinating enzymes (DUBs) are involved in this process (Amerik and Hochstrasser, 2004); DUBs maintain the cellular pool of free ubiquitin by processing ubiquitin precursors and recycling ubiquitin from poly-ubiquitinated substrates. Once polyubiquitinated, the substrate protein is recognized by the SQSTM1/p62, and other proteins of this class (Klionsky et al., 2016) and routed to the proteasome for degradation. The 26S proteasome has a typical barrel shape constituted by a large, multi-subunit protease complex: a 20S core complex with catalytic activity and a 19S regulatory complex, the cap. The cap receives the polyubiquitinated substrate, removes the poly-ubiquitin chain and induces its translocation into the 20S complex. Here, the substrate protein must enter the narrow central 20S cavity for the enzymatic degradation to small peptides. To this aim, folded proteins must be unfolded by the 19S subunit to reach a “linear” conformation. Thus, globular or aggregated proteins are not processed by the proteasome (Ciechanover and Brundin, 2003), and may even clog its catalytic core. Molecular chaperones and co-chaperones cooperating with the proteasomal-mediated degradation of ubiquitinated substrates include the already mentioned HSP70/HSP40 (now identified as HSPAs/DNAJs) and the HSP70/BAG1 complexes (Figure 1; Demand et al., 2001; Alberti et al., 2002; Kampinga and Craig, 2010; Kampinga and Bergink, 2016; Cristofani et al., 2017; Cicardi et al., 2018, 2019). In the latter case, the HSP70/CHIP complex, initially described as required for the substrate ubiquitination, can associate to BAG1, and together with SQSTM1/p62 it drives the ubiquitinated misfolded protein to proteasomal degradation.

(b) The lysosomal-mediated system collects proteins from various origins. The system is typically divided into microautophagy, chaperone-mediated autophagy (CMA), and macroautophagy (normally identified as autophagy). These systems are evolutionarily well-conserved processes required for the degradation of proteins or large cytosolic components *via* the lysosome (Mizushima et al., 2008). In the case of microautophagy, the cytosolic components are directly engulfed into lysosomes *via* an invagination of its membrane (Sahu et al., 2011). In CMA, only a specific subset of proteins can be processed: those containing a pentapeptide lysosome-targeting motif KFERQ or related consensus motifs (also generated by specific post-translational modifications; Orenstein et al., 2013; Kaushik and Cuervo, 2018; Kirchner et al., 2019); the sequence allows the direct translocation of cargo into lysosome. CMA requires the docking to the lysosomal receptor lysosome-associated membrane protein 2A (LAMP2A), as well as the protein unfolding by a chaperone complex containing HSC70, BAG1, HSC70-interacting protein (HIP), Hsp-organizing protein (HOP), and HSP40 (DNAJB1; Kampinga et al., 2009; Kampinga and Craig, 2010; Kampinga and Bergink, 2016). Instead, in macroautophagy (which the general term “autophagy” usually refers to), the cytosolic components are engulfed into the autophagosome, a double-membrane vesicle that then fuses with the lysosome, in order to deliver its content to the lysosome for degradation (Xie and Klionsky, 2007). Initially considered as a sort of non-specific degradation for long-lived proteins, organelles, or protein aggregates, it is now clear that autophagy is tightly regulated by several pro-autophagic factors (Mizushima et al., 2008; Sardiello et al., 2009). In this latter form of autophagy, it is also possible to distinguish between “in bulk” autophagy and selective autophagy. While “in bulk” autophagy is characterized by a very high clearance capability but is rather non-specific since it entraps large portion of cytoplasm, selective autophagy is highly specific and involves specific molecular regulators (Kaushik and Cuervo, 2018). Selective autophagy includes chaperone-assisted selective autophagy (CASA; Arndt et al., 2010; Kettern et al., 2011; Sarparanta et al., 2012; Ulbricht et al., 2013, 2015; Ghaoui et al., 2016; Sandell et al., 2016; Cicardi et al., 2019; Cristofani et al., 2019; Rusmini et al., 2019), organelles-specific types of autophagy (mitophagy, lysophagy, ribophagy, granulophagy, etc.), or processes aimed at removing large protein aggregates (aggrephagy; Nivon et al., 2012; Stürner and Behl, 2017; Aparicio et al., 2020).

CASA has attracted large attention in the field of NDs, specifically in MNDs, since this highly selective autophagy is based on the recognition of misfolded substrates by a heteromeric complex composed of a small HSP, the HSPB8, with its co-chaperone BAG3. Once the misfolded protein is bound to HSPB8/BAG3, the HSP70/CHIP dimer (already seen in the UPS pathway) can be recruited. Here, the misfolded protein is rapidly ubiquitinated by CHIP, allowing recognition by the autophagy receptor SQSTM1/p62 (and related proteins) and forming the CASA complex. Some studies include HSP40 or DNAJ proteins in this complex (Sarparanta et al., 2012; Sandell et al., 2016). In this context, the role of SQSTM1/p62 is different from that exerted in association with BAG1/HSP70/CHIP, which allows the use of the UPS pathway. When acting with HSPB8/BAG3/HSP70/CHIP, the SQSTM1/p62 protein interacts with the ubiquitinated misfolded proteins (or other cargoes) and the lipidated form of the microtubule-associated proteins 1A/1B light chain 3B (LC3-II) anchored to the autophagosome membrane. To allow SQSTM1/p62 and LC3-II-action, the CASA complex takes advantage of a dynein binding motif present in the BAG3 sequence. The CASA complex bound to dynein is transported along microtubules to the microtubule organizing center (MTOC). Ubiquitinated and SQSTM1/p62-positive misfolded proteins are concentrated at MTOC to form the aggresomes. Meanwhile, LC3-II decorated-autophagosomes are generated, allowing aggresome insertion into a nascent autophagosome. The autophagosome containing the CASA complex and the misfolded proteins fuses with the lysosome to allow the degradation of the engulfed material following the canonical autophagic pathway.

Selective autophagy is also involved in the degradation of damaged organelles like mitochondria and lysosomes. In mitophagy, the damaged mitochondria stabilize PINK1 on its outer membrane. PINK1 recruits E3-ubiquitin ligases, like Parkin, which amplify the ubiquitination of proteins in the outer membrane mediating recruitment of the autophagic receptors that interact with LC3-II present on the forming autophagosome membrane (Youle and Narendra, 2011). Some of the mitochondrial membrane proteins, like mitofusin, are polyubiquinated with K48 ubiquitin chains. These proteins are substrates of VCP/p97, an AAA^+^ ATPase, that segregates these proteins from the mitochondria membrane and promotes their degradation *via* UPS. The removal of these proteins is necessary for mitochondria degradation (Tanaka et al., 2010; Tanaka, 2010; Kimura et al., 2013). In lysophagy, ruptured lysosomes expose galectins (Gal-3, Gal-8) as damage signals. Gal-8 is directly recognized by autophagy receptors, while Gal-3 recruits and binds TRIM16. Gal-3/TRIM16 complex promotes ubiquitination of lysosomal proteins and recruits autophagy initiation factors to trigger local phagophore formation (Thurston et al., 2012; Chauhan et al., 2016). Moreover, K63-ubiquitinated proteins recruit autophagy receptors, while K48-ubiquitinated proteins are targeted by VCP/p97 to UPS degradation. VCP/p97 recruitment to lysosome membranes and functioning are mediated by its cofactors and adaptors YOD1, UBXD1, and PLAA. The removal of K48 polyubiquitinated proteins is a critical step to promote lysosome degradation (Fujita et al., 2013; Akutsu et al., 2016; Papadopoulos et al., 2017).

### The Unfolded Protein Response (UPR) and the Endoplasmic Reticulum-Associated Degradation (ERAD)

UPR and ERAD are two other key pathways devoted to the PQC in cells. UPR is typically activated in the presence of an abnormal excess of misfolded proteins, while ERAD mediates their degradation by taking advantage of the cytosolic proteasome mentioned above. In fact, the accumulation of misfolded proteins in the endoplasmic reticulum (ER) activates the UPR. This action is mediated by three different “sensors”—inositol requiring enzyme 1 (IRE1α), PKR-like endoplasmic reticulum kinase (PERK), and activating transcription factor 6 (ATF6; Hetz, 2012)—that signal to dedicated pathways to stimulate either protein folding or protein degradation. During this process, ribosomes are forced to attenuate protein translation. ERAD has a specific function in PQC system since the ER is a major site for protein folding. When aberrant ER-resident proteins are processed by ERAD, they are released into the cytosol for proteasomal (when these are still soluble) or for autophagic clearance (when they are in an aggregated form; Hetz, 2012). Even in the case of ERAD, the proteins are ubiquitinated by specific E3-ubiquitin ligases, like HRD1 in the SEL1L-HRD1 protein complex (where SEL1L acts as a cofactor). Ubiquitinated misfolded proteins can be “retro-translocated” or “dislocated” (extracted) from the ER membrane and transported to the cytosol mainly by the activity of VCP/p97. VCP/p97 in complex with UFD1-NPL4 first binds HRD1 and the ubiquitinated proteins, then addresses substrates to the proteasome *via* shuttle cofactors (Ye et al., 2005; Senft and Ronai, 2015). Even in the case of the UPR-ERAD, a central role is played by an HSP70, BiP (or HSPA5 or GRP-78), which has low intrinsic ATPase activity, enhanced by co-chaperones of the DNAJ-proteins (the same class of the HSP40, like ERdj4 or DNAJB9). In addition to protein folding, the ER controls the Ca^2+^ homeostasis, being the major intracellular Ca^2+^ reservoir (Hetz and Mollereau, 2014). When misfolded proteins accumulate in the ER, the depletion of ER Ca^2+^ impacts on cell activity and enhances stress. Store-operated Ca^2+^ influx is activated in these conditions to assure the replenishment of Ca^2+^ levels (Szegezdi et al., 2006). If ER stress is prolonged, the ability of the UPR to restore ER homeostasis is reduced and this may cause ER stress-induced apoptosis by activation of caspase 12 (Yoneda et al., 2001). Once activated, the UPR-ERAD converges on the proteasome or to autophagy; therefore, in this review we will only focus on the proper degradative pathways. Details on UPR-ERAD can be found elsewhere (for an extensive review see Hwang and Qi, 2018).

### Release Mediated by Extracellular Vesicles

Emerging data strongly suggest that the extracellular secretion may also play an important role in the maintenance of intracellular protein homeostasis by cooperating with or even being a part of the PQC system (Desdín-Micó and Mittelbrunn, 2017; Xu et al., 2018; Guix, 2020). In fact, it has been found that several NDs-related proteins are secreted in double membrane spherical particles known as extracellular vesicles. This is the case for the amyloid-beta peptide and tau/phosphorylated tau for Alzheimer’s disease (Pérez et al., 2019), alpha-synuclein for Parkinson’s disease (Longoni et al., 2019), misfolded/mutant SOD1, TDP-43 and its pathological-related C-terminal fragments (of 35 kDa and 25 kDa) and FUS for ALS (Basso and Bonetto, 2016; Iguchi et al., 2016; Hanspal et al., 2017; Sproviero et al., 2018), and progranulin, TDP-43, and C9orf72 DPRs for FTD and ALS-FTD (Benussi et al., 2016; Iguchi et al., 2016; Westergard et al., 2016). The extracellular vesicles are heterogeneous in size and are mainly classified into three different types: exosomes, microvesicles, and apoptotic bodies. These vesicles differ for size, proteins, and lipids composition and intracellular origin. In fact, exosomes are secreted membrane vesicles (approximately 30–120 nm in diameter) formed intracellularly and released from exocytosis of multivesicular bodies, whereas apoptotic bodies (approximately 1,000–4,000 nm in diameter) are released by dying/apoptotic cells. Microvesicles (approximately 200–1,000 nm in diameter) are shed from cells by an outward protrusion (or budding) of the plasma membrane followed by fission of their membrane stalk (for a detailed review see Akers et al., 2013; Colombo et al., 2014; van Niel et al., 2018). A tight connection between PQC and extracellular vesicles is particularly true for exosomes (Xu et al., 2018). As stated above, exosomes are intraluminal vesicles of the endosomal compartment that maturate into a structure called the multivesicular body after a very dynamic process. The multivesicular body may release its content into the lysosome for degradation or, under certain conditions, it may fuse with the plasma membrane and secrete its intraluminal vesicles, the exosomes. Interestingly, components of the CASA complex may also affect/take part in extracellular vesicles pathway: for example, STUB1/CHIP deficiency resulted in an increased secretion of small extracellular vesicles that are enriched in ubiquitinated and/or undegraded proteins and protein oligomers (Ferreira et al., 2019), and BAG3 is found to be involved in the exosome secretion of mutant Huntingtin upon proteasome blockade (Diaz-Hidalgo et al., 2016). These evidences suggest that extracellular vesicles have to be considered as new actors in the proteostasis scenario, together with chaperones and the degradative systems.

## How the Protein Quality Control System Protects Against Misfolded Protein Toxicity in SBMA and ALS

Data collected over the last 30 years suggest that ARpolyQ and several ALS-associated proteins (listed in Table 1) may lead to PQC system alterations (Kabashi and Durham, 2006; Voisine et al., 2010; Rusmini et al., 2016, 2017; Cristofani et al., 2018, 2019). At the same time, the boost of key proteins involved in PQC system regulation is protective in SBMA and ALS (Waza et al., 2006; Yu et al., 2011; Giorgetti et al., 2015; Crippa et al., 2016b; Rusmini et al., 2016, 2017, 2019, 2020; Cristofani et al., 2018, 2019; Mandrioli et al., 2019).

**Figure 2:**
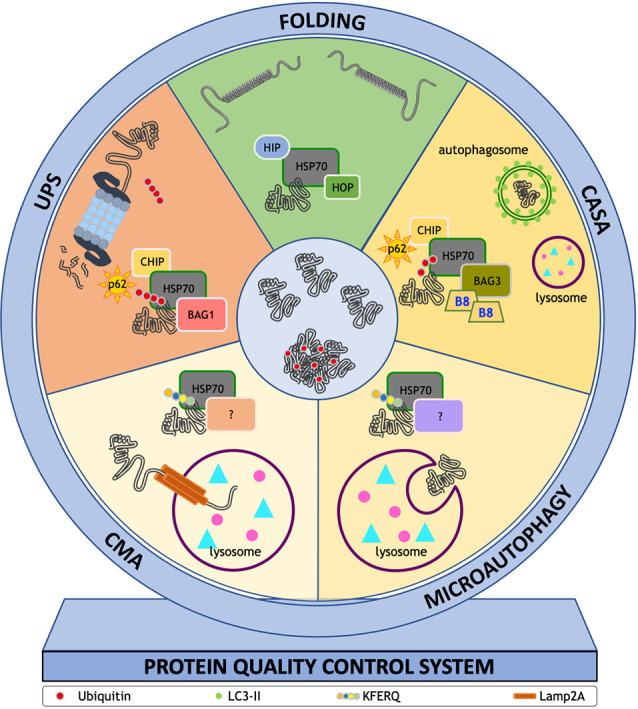
ThePQC system. The fate of misfolded proteins is finely tuned by the PQC system. This system is centered on a group of chaperones and co-chaperones assisting proteins to reach their correct conformation or directing proteins to degradative systems. Each pathway needs specific proteins that assist the action of HSP70: (i) HSC70 interacting protein 1 (HIP) and HSP70-HSP90 organizing protein (HOP) in the folding process; (ii) sequestosome 1 (SQSTM1/p62), E3-ubiquitin ligase C-terminus HSP70 interacting protein (CHIP), BAG family molecular chaperone regulator 3 (BAG3) and heat shock protein B8 (HSPB8, B8 in figure) in chaperone assisted selective autophagy (CASA); (iii) Lysosome-associated membrane glycoprotein 2 (Lamp2A) in chaperone mediated autophagy (CMA); and (iv) SQSTM1/p62, CHIP, BAG family molecular chaperone regulator 1 (BAG1) in ubiquitin proteasome system (UPS). The HSP70 interactors at lysosome membrane remain to be determined [indicated in figure as (?)] even if coimmunoprecipitation and colocalization studies identified HSP90, HSP40, HOP, HIP, and BAG1. Their role in CMA and microautophagy remains to be determined.

Figure 2 summarizes how all the PQC system components work synergistically to prevent misfolded protein accumulation in these diseases.

### Folding Process

The first line of PQC system intervention on the misfolded protein is an attempt to restore the proper protein folding. Even if the folding process is well-understood, many questions still remain open in the case of disease-associated misfolded proteins; in particular, to what extent the refolding of a protein may occur after the first aggregation steps. The main actors in the folding process are the HSP70s (also named HSPAs), which are similar to nanomachines capable of switching conformation using hydrolysis of ATP (Figure 1). This allows HSP70 to change conformation in order to assist protein folding, disaggregation, and degradation (see Kampinga and Craig, 2010; Kampinga and Bergink, 2016 for an extensive review). HSP70 is a hub that requires the assistance of HSP40s (or DNAJ proteins) in order to recognize the protein to be folded, and of NEFs, like the BAGs, which exchange ADP/ATP during the hydrolytic process (Sondermann et al., 2001; Rauch and Gestwicki, 2014).

Misfolded proteins responsible for SBMA and ALS are able to alter this finely-tuned process. These misfolded proteins escape the correct folding and expose unstructured domains highly prone to aggregate. Such domains are present in the ARpolyQ in its poorly structured N-terminus containing the polyQ stretch, in the prion-like domains of TDP-43 and FUS proteins, and maybe also in the five DPRs derived from the C9orf72 mRNA, which do not possess tertiary structures. These unstructured domains may clamp together in a liquid-liquid partitioning of phases, forming membraneless organelles attracting other compatible molecules (e.g., RNAs or proteins which normally interact with these unstructured proteins). The mutations in these proteins greatly enhance their capability to generate liquid-liquid intracellular compartments, which soon after their formation may mature into aggresomes, stable aggregates, and even insoluble inclusions trapping specific intracellular factors (Molliex et al., 2015; Patel et al., 2015; Ganassi et al., 2016; Alberti et al., 2017; Boeynaems et al., 2017; Mackenzie et al., 2017; Mateju et al., 2017; Marrone et al., 2018). Specific proteins are known to accelerate the conversion of the aggregates formed after phase separation into stable insoluble aggregates. Conversely, chaperones and co-chaperones may prevent this conversion, delaying the maturation into stable structures and facilitating the disassembling of the newly formed membraneless organelles. This activity of chaperones and co-chaperones should permit the refolding process of a misfolded protein even after its entrapping in the aggresomes when they are still dynamic (Jaru-Ampornpan et al., 2013; Mattoo and Goloubinoff, 2014; O’Driscoll et al., 2015; Zaarur et al., 2015; Mathew and Stirling, 2017; Kitamura et al., 2018; Alexandrov et al., 2019).

### Alterations of UPS in SBMA and ALS

Evidence suggests that ARpolyQ, SOD1, TDP-43, and its ALS associated fragments, as well as other ALS-proteins, including at least one out of five DPRs of C9orf72, are processed *via* the proteasome (Rusmini et al., 2007, 2010, 2011, 2013, 2019; Sau et al., 2007; Crippa et al., 2010b, 2016a; Onesto et al., 2011; Cristofani et al., 2017, 2018; Cicardi et al., 2018, 2019). However, the large amounts of misfolded proteins formed when specific gene mutations occur may overwhelm the UPS capability to degrade them efficiently. This process is accentuated in aged cells in which the chaperone and UPS activities are reduced (Ciechanover and Brundin, 2003; Terry et al., 2004, 2006; Wang K. et al., 2018; Hegde et al., 2019). It is also possible, as in the case of the elongated polyQ tract of the AR, that the proteasome proteolytic capability is unable to digest the long polyQ sequence since no consensus cleavage sites for its enzymatic activity are present between the Qs; thus, long uninterrupted polyQ size might block the narrow catalytic site, where only a single protein can enter and be degraded. We showed that while the wtAR with a 23Q stretch can be cleared *via* the proteasome even in presence of androgens (Rusmini et al., 2007), the mutant ARpolyQ may impair the UPS function; in fact, by expressing the ARpolyQ in basal condition (absence of androgens), we noted an accumulation of the proteasome activity reporter GFP-CL1 (GFPu) as an indication that the elongated polyQ is poorly processed by the UPS and interferes with the activity of this degradative pathway. Interestingly, the inactivated ARpolyQ does not play toxicity in all cell models tested. Surprisingly, when the ARpolyQ is activated by androgens (which bound at the AR C-terminus), the protein is thought to acquire toxic conformations (Stenoien et al., 1999; Simeoni et al., 2000; Piccioni et al., 2002; Poletti, 2004), but the UPS is fully functional (Rusmini et al., 2007) since the GFPu reporter is fully degraded by the UPS. An explanation for this unexpected phenomenon is that by inducing the ARpolyQ toxic conformation, androgens also induce its misfolding (possibly *via* a phase partitioning phenomenon; Eftekharzadeh et al., 2019; Escobedo et al., 2019) and sequestration into subcellular compartments (the aggregates), protecting the cell from this dangerous protein conformation (Rusmini et al., 2007, 2010). The formation of aggregates acts as a sink that permits UPS desaturation from the excess of “free” polyQ to be processed. Meanwhile, aggregates might stimulate autophagy for ARpolyQ clearance (see below). It is thus expected that also autophagy alterations might contribute to the accumulation of stable insoluble ARpolyQ aggregates in cells (Rusmini et al., 2013; Giorgetti et al., 2015; Cristofani et al., 2017; Cicardi et al., 2019). As will be discussed below, the potentiation of CASA restores normal ARpolyQ clearance. A similar UPS role has been found involved in the clearance of ALS-misfolded proteins. Mutant SOD1 is mainly cleared by the UPS, and its pharmacological inhibition with MG132 results in an accumulation of ubiquitin-positive SOD1 aggregates in cells (Crippa et al., 2010a,b, 2016a; Cicardi et al., 2018). This aggregated SOD1 is poorly removed by autophagy but, as seen for ARpolyQ, the induction of CASA restores complete clearance of aggregating mutant SOD1 (Crippa et al., 2010a,b, see below). TDP-43 and its 35 kDa and 25 kDa TDP-43 fragments follow the same route of degradation identified for inactive ARpolyQ and mutant SOD1 (Crippa et al., 2016a; Cicardi et al., 2018, 2019). Even in this case, UPS inhibition results in an accumulation and mislocalization of TDP-43 and fragments, aside from the 25 kDa TDP-43 fragment. CASA induction reverts also this phenotype (Crippa et al., 2016a; Cicardi et al., 2018). It is unclear whether autophagy defects play a major role in the accumulation of these TDP-43-related aberrant species and this may underline differences in the type of toxicity exerted by these MNDs proteins. Recent works have shown that TDP-43 inclusions and TDP-43 hyperphosphorylation (typical hallmarks of ALS-motor neurons) are also present in muscles in sALS patients. This discovery raised a question: whether TDP-43 misfolded species could accumulate and exert toxicity in muscle cells. We found that the insoluble TDP-43 fragments also accumulate in muscle C2C12 cells, but their aggregation is reverted by tuning the expression of key components of the CASA complex. Whether the accumulation of these fragments in muscle tissue is causative of muscle atrophy is yet to be elucidated (Cicardi et al., 2018).

Also, the C9orf72 DPRs degradation is mediated by UPS and autophagy, even with different behavior of the five DPRs, since only the polyGP is efficiently degraded by the UPS (Cristofani et al., 2018). PolyGP is also degraded *via* autophagy that is able to efficiently remove polyPA, polyGR, and polyGA (Cristofani et al., 2018). Conversely, only the polyPR seems to be resistant to both degradative systems in basal condition. The reasons for these differences are still unclear, but CASA activation prevents the accumulation of all five DPRs (Cristofani et al., 2018), as will be described below.

Collectively, these data suggest that several MND-associated misfolded proteins can be cleared by the UPS system, possibly in a monomeric state. It is expected that UPS overwhelming will result in an accumulation of these species that, once concentrated in specific subcellular compartments (liquid-liquid, aggresomes, etc.), may reversibly aggregate. This mechanism might protect from misfolded protein toxicity since these species are sequestered, limiting their potential toxicity. If the accumulation persists, the aggregates may mature to more stable and potentially toxic species, and thus must be removed using alternative strategies by the cells.

### Alteration of Autophagy and CASA in SBMA and ALS

Alteration of autophagy has been reported in animal and cell models of SBMA (Montie and Merry, 2009; Yu et al., 2011; Doi et al., 2013; Rusmini et al., 2013, 2015, 2019; Chua et al., 2014; Cortes et al., 2014b; Thellung et al., 2018; Cicardi et al., 2019) and ALS (Kabuta et al., 2006; Morimoto et al., 2007; Li et al., 2008; Wang et al., 2012; Crippa et al., 2013; Xiao et al., 2015; Evans and Holzbaur, 2019; Nguyen et al., 2019). Despite this, the complexity of the autophagic pathway makes it difficult to fully understand which level of this multistep process is affected by the presence of misfolded proteins. It is evident from experimental data that autophagy activation has a beneficial role in disease since pharmacological or genetic induction of autophagy ameliorates disease phenotype (e.g., delaying disease onset, slowing down its progression or ameliorating motor behavior; Montie et al., 2009; Wang et al., 2012; Castillo et al., 2013; Kim et al., 2013; Tohnai et al., 2014; Zhang et al., 2014; Giorgetti et al., 2015; Li et al., 2015; Perera et al., 2018; Wang Y. et al., 2018; Rusmini et al., 2019). Unfortunately, not all studies agree with these observations (Zhang et al., 2011). By focusing on CASA, which has already been mentioned above, it must be noted that HSP70 chaperones and others require the assistance of co-chaperones, like the member of the NEF family (Kampinga and Craig, 2010), which includes the BAGs (Takayama and Reed, 2001). Cells utilize different BAGs to route misfolded proteins either to the UPS or to autophagy (Figure 1). BAG1 associates to HSP70 and CHIP to route misfolded proteins to the UPS, while BAG3, in association with HSPB8, interacts with HSP70/CHIP to route misfolded proteins to autophagy (Figure 2). This allows to select which pathway has to be followed by misfolded proteins to be efficiently cleared from cells; the perturbation of this equilibrium may result in misfolded proteins accumulation (Cristofani et al., 2017, 2019; Rusmini et al., 2017). The importance of the CASA complex in cell protection against proteotoxicity is underlined by the fact that mutations in the genes coding for almost all components of the CASA complex have been associated with human diseases. Indeed, mutations in HSPB8 cause diseases of motoneurons and/or muscle cells [Charcot-Marie-Tooth (CMT) type 2L disease, hereditary distal motor neuropathy type II (dHMN-II), or distal myopathy; Fontaine et al., 2006; Irobi et al., 2010; Ghaoui et al., 2016; Al-Tahan et al., 2019]. Mutations in BAG3 are causative of dilated cardiomyopathy (Arimura et al., 2011), muscular dystrophy (Selcen et al., 2009), giant axonal neuropathy, and late-onset axonal CMT neuropathy (Jaffer et al., 2012; Shy et al., 2018). Interestingly, three BAG3 mutations involve the Pro209 residue (Pro209Leu, Pro209Ser, Pro209Glu), which falls in one of the two HSPB8-interacting Ile-Pro-Val (IPV) motifs. These Pro209 mutants still retain the ability to bind to all CASA members but they impair HSP70 client processing, and they accumulate at the aggresome preventing target protein degradation and sequestering CASA members (Meister-Broekema et al., 2018; Adriaenssens et al., 2020). Mutation in STUB1/CHIP have been found in Gordon Holmes syndrome (multisystemic neurodegeneration; Hayer et al., 2017) and more recently in SCA48 (Genis et al., 2018), and a destabilized CHIP (linked to six different variants) is present in SCA16 (Pakdaman et al., 2017; Kanack et al., 2018); also, a missense mutation in the CHIP-ubiquitin ligase domain was reported as the cause of a form of spinocerebellar autosomal recessive 16 (SCAR16; Shi et al., 2013, 2018). Mutations of the SQSTM1/p62, which recognizes the CHIP-ubiquitinated cargo inside the CASA complex (some authors include it as a member of this complex), are responsible for fALS (Fecto et al., 2011; Teyssou et al., 2013). Of note, it has been suggested that in skeletal muscle, DNAJB6 of the DNAJ/Hsp40 family (HSP70 co-chaperones) suppresses aggregation of misfolded proteins involved in NDs (Hageman et al., 2010) and participates to the formation of the CASA complex (Sarparanta et al., 2012). Interestingly, a mutation in *DNAJB6* causes Limb-girdle muscular dystrophies (LGMDs), characterized by aggregates of DNAJB6 sequestering CASA complex proteins (Sandell et al., 2016).

The CASA complex is involved in mutant SOD1-associated fALS (Crippa et al., 2013). Indeed, mutant SOD1 induces a robust autophagic response both in the spinal cord and in muscle. BAG1, BAG3, HSPB8, LC3, and SQSTM1/p62 are significantly upregulated in mutant SOD1 transgenic ALS mice at the symptomatic stage (16 weeks). Notably, the autophagic response is much higher in muscle than in spinal cord, supporting the absence of high molecular weight insoluble species of mutant SOD1 in muscle; this also suggests that the toxicity exerted by mutant SOD1 in muscle cells is probably not related to the classical mechanism of intracellular protein aggregation (Galbiati et al., 2012, 2014). Interestingly, an analysis performed in SBMA knock-in mouse model revealed that the CASA complex is highly upregulated in skeletal muscle after disease onset, while no variations were observed in the spinal cord. In fact, HSPB8 and BAG3 mRNA and protein levels are increased in SBMA mice at the symptomatic stage compared to control, as well as the co-chaperone BAG1, involved in routing misfolded proteins to UPS. The increased BAG3 to BAG1 ratio suggested that autophagy is the main proteolytic pathway activated in muscle tissue during SBMA progression and CASA complex is involved in reducing ARpolyQ toxicity in skeletal muscle, which is a primary site of SBMA pathogenesis (Rusmini et al., 2015). HSPB8 seems to be a limiting factor for the CASA complex (Crippa et al., 2010a,b). HSPB8 overexpression rescues from protein accumulation and aggregation of mutant SOD1 and TDP-43 in cell models of ALS (Crippa et al., 2010a,b), while its silencing has opposite effects favoring misfolded proteins accumulation in motor neurons (Crippa et al., 2010a,b). Overlapping data were obtained with other misfolded proteins implicated in Alzheimer’s disease, Parkinson’s disease, a form of spinal cerebellar ataxia, (SCA3), SBMA, fALS, and FTD. In fact, HSPB8 enhances the autophagy clearance of beta-amyloid, alpha-synuclein (α-syn), the polyQ proteins huntingtin, ataxin-3, and ARpolyQ, as well as all five DPRs from the *C9orf72* mRNA (Chávez Zobel et al., 2003; Wilhelmus et al., 2006; Carra et al., 2008a,b; Crippa et al., 2010b, 2016a; Bruinsma et al., 2011; Seidel et al., 2012; Rusmini et al., 2013, 2016; Giorgetti et al., 2015; Cicardi et al., 2018), while HSPB8 downregulation has the opposite effects (Crippa et al., 2010b, 2016a,b; Rusmini et al., 2013; Cristofani et al., 2017).

Since HSPB8 may be a limiting factor of CASA complex activity and its overexpression is sufficient to restore autophagy, it is clear that this protein represents a valid therapeutic target for these NDs. It has been demonstrated that HSPB8 expression is induced by estrogens and other selective estrogen receptor modulators (SERMs; Sun et al., 2007; Piccolella et al., 2014, 2017; Meister-Broekema et al., 2018), and this could help to explain why gender differences occur in the appearance of several NDs (Vegeto et al., 2020). Recently, we set up a high throughput screening (HTS) using a reporter luciferase gene under the transcriptional control of the human HSPB8 promoter. With this system, we found that colchicine [a Food and Drug Administration (FDA)- and European Medicine Agency (EMA)-approved drug] stimulates HSPB8 expression and enhances the autophagy clearance of the insoluble TDP-43 species (Crippa et al., 2016a) in models of ALS. The drug is presently in phase II clinical trial for ALS (Mandrioli et al., 2019). Other HSPB8 inducers are some disaccharides, like trehalose, melibiose, or lactulose (Rusmini et al., 2013, 2019; Giorgetti et al., 2015). Trehalose has been tested in mouse models of Huntington’s disease, ALS, Parkinson’s disease, Alzheimer’s disease, succinate semialdehyde dehydrogenase deficiency, and oculopharyngeal muscular dystrophy, and found to be capable of ameliorating disease course and symptomatology (Tanaka et al., 2004; Davies et al., 2006; Rodriguez-Navarro et al., 2010; Perucho et al., 2012; Schaeffer and Goedert, 2012; Castillo et al., 2013; Du et al., 2013; Sarkar et al., 2014; Zhang et al., 2014; He et al., 2016). The mechanism of action of trehalose and its analogs, melibiose and lactulose, was recently uncovered. These disaccharides induce transient lysosomal permeabilization and possibly calcium release from lysosomes. These events trigger the Transcription Factor EB (TFEB) pathway, mediated by the calcium-dependent phosphatase PPP3/calcineurin, which dephosphorylates TFEB. Trehalose-activated TFEB migrates into the nucleus where it acts on CLEAR responsive elements to enhance the expression of genes controlling autophagy and lysosomal biogenesis. With this mechanism trehalose/TFEB-mediated activation of autophagy promotes the clearance of damaged lysosomes through lysophagy, but in parallel exerts neuroprotection by promoting the degradation of mutant and misfolded proteins from neurons (Rusmini et al., 2019).

Both colchicine and trehalose also induce BAG3 expression (Lei et al., 2015; Crippa et al., 2016b), indicating that these compounds may act *via* a general potentiation of CASA. Other drugs have been found able to stimulate BAG3 expression (e.g., proteasome inhibitors, TNF-related apoptosis-inducing ligand, fludarabine, cytosine arabinoside, and etoposide) but unfortunately, these drugs are used in chemotherapy with relevant side effects, and are thus not suitable for NDs (Romano et al., 2003; Chiappetta et al., 2007; Rapino et al., 2014). However, they might serve as molecule templates for the development of safer and better tolerated derivatives.

### Alteration of CMA in SBMA and ALS

Nothing is known so far about the involvement of CMA in the degradation of ARpolyQ in SBMA. Instead, recent data suggest that CMA may play a role in NDs, including ALS (Ormeño et al., 2020). Indeed, CMA is essential in Parkinson’s disease where its dysregulation modifies the onset or progression of the disease (Arias and Cuervo, 2011; Cuervo, 2011; Alfaro et al., 2018; Kaushik and Cuervo, 2018). Alpha-synuclein protein, leucine-rich repeat kinase 2 (LRRK2), Parkinson disease protein 7 (PARK7), and DJ-1, as well as myocyte-specific enhancer factor 2D protein (MEF2D), which are dysregulated or mutated in Parkinson’s disease, are CMA substrates (Vogiatzi et al., 2008; Yang et al., 2009; Arias and Cuervo, 2011; Cuervo, 2011; Orenstein et al., 2013; Murphy et al., 2015; Alfaro et al., 2018; Kaushik and Cuervo, 2018). Alzheimer’s disease is also associated with CMA since the beta-amyloid peptide (Aβ), the microtubule-associated protein Tau or the Regulator of calcineurin 1 (RCAN1) are involved in Alzheimer’s disease and are dysregulated when CMA is altered (Liu et al., 2009; Wang et al., 2009, 2010; Park et al., 2016). CMA also plays a role in Huntington’s disease (Koga et al., 2011; Qi et al., 2012), and mutant huntingtin can sequester LAMP2A and HSC70, two major players of CMA (Alfaro et al., 2018).

In ALS, CMA has been involved in TDP-43 metabolism (Huang et al., 2014). These data were recently corroborated by a study of the group of Budini et al., who pointed out that also TDP-43 can be a CMA substrate (Ormeño et al., 2020). This study started from the observation that TDP-43 contains a KFERQ-like domain, the consensus sequence that allows the interaction with HSC70 (Huang et al., 2014); mutation in this domain blocks the ubiquitin-dependent binding of TDP-43 with HSC70. Other authors have shown that LAMP2A downregulation induces the intracellular accumulation of the ALS-associated TDP-43 fragments of 35 and 25 kDa (Huang et al., 2014), and TDP-43 can also be forced to be degraded *via* CMA (Tamaki et al., 2018). Ormeño et al. (2020) showed *in vitro* that a recombinant form of TDP-43 is processed by isolated rat liver lysosomes, a process that can be reduced by competition with the GAPDH protein, a typical CMA substrate. Endogenous TDP-43 accumulates in CMA^+^ lysosomes of the brain (Ormeño et al., 2020). By using an artificial TDP-43 aggregate-prone protein, Ormeño et al. (2020) demonstrated its interaction with HSC70 and LAMP2A, which causes an upregulation of CMA activity and lysosomal damage. These data open up the question of how CMA is involved not only in the few fALS forms associated with mutations of TDP-43, but also in the vast majority of sALS forms characterized by an intense mislocalization and accumulation of TDP-43 in affected neuronal and motor neuronal cells of ALS patients. By analyzing the two CMA regulators (LAMP2A and HSC70) in peripheral blood mononuclear cells (PBMCs) of ALS patients, it was found that the levels of the lysosome receptor LAMP2A were similar in control and ALS PBMCs, while the expression of the cytosolic chaperone HSC70 was found reduced, but the total amount of insoluble TDP-43 protein was found increased and accompanied by aberrant intracellular localization (Arosio et al., 2020). In parallel, HSC70 downregulation in human neuroblastoma cells correlates with the increased accumulation of the TDP-43 protein (Arosio et al., 2020). These data are in line with experimental observation showing that HSC70 is reduced in motor neurons of TDP-43-based ALS fly models, as well as in iPSCs C9orf72 models differentiated to motor neurons (Coyne et al., 2017). In addition to these observations, in ALS-PBMCs, the ratio of the expression levels and protein of BAG1 and BAG3, which determines the equilibrium between proteasome and autophagy (including CASA), was also found altered (Arosio et al., 2020). Thus, even if CMA is not directly affected in ALS-PBMCs, the reduction of the CMA regulator HSC70 may be involved in ALS pathogenesis.

### Alteration of UPR-ERAD in SBMA and ALS

As mentioned above, proteasome and autophagy work together in response to proteotoxic stimuli. Both pathways are also involved downstream in the UPR occurring in the ER. The UPR, activated in the ER lumen, generates a transient translational inhibition along with the induction of chaperones and the stimulation of the degradative pathways. Misfolding proteins here are identified by BiP/GRP78 (an HSP70), which assists the ERAD, also activating PERK and IRE1. The PERK receptor attenuates translation in response to UPR involving oligomerization and autophosphorylation of PERK with eIF2alpha phosphorylation. In parallel, the transcription factor XBP1 activated by alternative splicing induces UPR stress genes, while cleaved activated ATF6 exits the ER and moves to the nucleus to stimulate other UPR genes. Collectively, this restores ER activities: in SBMA, an ARpolyQ N-terminal fragment activates ER stress-inducible promoter *via* ATF6, IRE1, and PERK. Indeed, ARpolyQ toxicity is enhanced by ATF6 blockage and reverted by ATF6 overexpression. Also, stimulation of PERK increases ARpolyQ toxicity (Thomas et al., 2005). Thus, ARpolyQ induces UPR, while UPR stimulation is protective in SBMA (Rusmini et al., 2016). In a SBMA knock-in mouse model, the downregulation of transcription factor C/EBP homologous protein (CHOP), involved in UPR-ERAD, worsened muscle atrophy (Yu et al., 2011). In parallel, in mouse embryonic stem cells (ESCs), ARpolyQ inclusions sequester CHIP and BiP/GRP78, inducing ER stress and apoptosis. UPR was found with the induction of the ER chaperones BiP/GRP78 and GRP94 and the stress markers ATF6, phosphorylated PERK, GADD153/CHOP, and spliced XBP-1. Notably, BiP/GRP78 overexpression reverted this phenotype, while BiP/GRP78 downregulation had the opposite effect (Yang et al., 2013). As mentioned above, ER stress and Ca^2+^ homeostasis are tightly connected. In mouse model of SBMA (Sopher et al., 2004; Malik et al., 2011; Montague et al., 2014) alteration of Ca^2+^ homeostasis has been reported in embryonic motor neurons in response to ER stress causing ER-stress-induced apoptosis (Montague et al., 2014). ARpolyQ specifically depleted ER Ca^2+^ levels and the store-operated Ca^2+^ influx (Hetz and Mollereau, 2014; Tadic et al., 2014), possibly *via* the reduction of the sarcoendoplasmic reticulum Ca^2+^ ATPases (SERCA) 2b pump activity. This pump allows ER Ca^2+^ re-uptake (Foradori and Handa, 2008), and its dysregulation activates caspase 12 (Montague et al., 2014). Thus, ER stress is also involved in SBMA pathogenesis and may represent an additional therapeutic target for this disease.

ER morphology alterations occur both in ALS patients and ALS mouse models (Dal Canto and Gurney, 1995; Dal Canto, 1995; Oyanagi et al., 2008; Lautenschlaeger et al., 2012), possibly because of protein accumulation in ER causing ER stress (Sasaki, 2010). Also, the Golgi apparatus is affected in ALS (Fujita et al., 2000; Stieber et al., 2000). Mutant SOD1 inclusions in ER are positive for BiP/GRP78 and calnexin (Wate et al., 2005; Kikuchi et al., 2006), while some ER chaperones are upregulated in ALS patients and mice (Atkin et al., 2006). Notably, mutant SOD1 specifically binds Derlin-1, which controls the ERAD machinery, and triggers ER stress-induced apoptosis (Nishitoh et al., 2008). ER stress in ALS may also result from altered ER calcium homeostasis (Grosskreutz et al., 2010) or by ER-mitochondria calcium cycle unbalance (Damiano et al., 2006; Grosskreutz et al., 2010; Jaronen et al., 2014). In addition, ATF6, phospho-PERK, and phospho-eIF2α are elevated in ALS mice and cell models (Atkin et al., 2006, 2008; Saxena et al., 2009). In the spinal cord of ALS patients and mice, IRE1 is increased (Atkin et al., 2006, 2008) and its phosphorylated form correlated with spliced XBP1 in ALS mice (Kikuchi et al., 2006). Notably, autophagy is induced in double knockout/transgenic mice with mutant G86R-SOD1 and XBP1 blockage (Hetz et al., 2008; Hetz, 2012; Hetz and Mollereau, 2014), suggesting that autophagy may serve to protect when UPR/ERAD fails. A recent study performed by the group of de Belleroche suggests that at least 40 different target genes, associated with ERAD and regulated by XBP1 or ATF6, are altered in spinal cord specimens from ALS patients; this is paralleled by severe alterations and activation of the IRE1α-XBP1 and ATF6 pathways (Montibeller and de Belleroche, 2018). Among these genes, co-chaperones of the DNAJ family (*DNAJB9* and *DNAJC10*) modulating HSPA5 (BiP/Grp78, which is the only HSP70 in the ER; Kampinga and Bergink, 2016) were increased in this dataset. Both DNAJB9 and DNAJC10 are involved in ERAD (Behnke et al., 2015) and may suppress cell death induced by ER stress (Kurisu et al., 2003). As occurs in SBMA, misfolded proteins also impact ERAD-UPR in ALS, suggesting that similar strategies based on the reinforcement of this pathway can contribute to restore protein homeostasis in affected cells.

## Conclusions

In conclusion, data accumulated over the past 30 years have suggested that specific proteins cause MNDs by triggering aberrant responses in neurons and other cells involved in this group of diseases. The alteration of the PQC system is presently thought to be one of the major factors responsible for both the onset and progression rate of the disease. PQC systems failure could be directly associated with a mutant protein involved in one of the PQC pathways, or indirectly associated with effects caused by the overproduction of misfolded proteins that saturate or impair the PQC system activity. This leads to a reduced PQC potential to maintain the proper cellular homeostasis, especially during cell stresses. Notably, this system is presently considered a potential druggable target, since it provides huge numbers of players with activity that can be pharmacologically or genetically enhanced or modulated. Indeed, several of the cooperative factors playing a role in the PQC system can be specifically induced or downregulated, allowing the potentiation of a single arm of this defense mechanism. In many cases, the restoration of the proper function of one PQC arm has positive effects on the other arms of the system; they together provide a redundant mechanism capable of efficiently clearing most of the aberrant aggregating proteins, thus reducing cell death. Different approaches aimed to potentiate one or more arms of the PQC system have already been preclinically tested and are under investigation in clinical trials. Hopefully, these approaches will identify new treatments to counteract neurodegeneration in MNDs.

## Author Contributions

RC, MG, VC, PR, and AP designed and wrote the manuscript and critically discussed all sections of the minireview. In addition, RC prepared the figures. MEC, VF, BT, EC, MC, EM, and MP critically revised the manuscript and the figures. All authors have provided final approval of the version to be published.

## Conflict of Interest

The authors declare that the research was conducted in the absence of any commercial or financial relationships that could be construed as a potential conflict of interest.
